# A Novel Resveratrol Based Tubulin Inhibitor Induces Mitotic Arrest and Activates Apoptosis in Cancer Cells

**DOI:** 10.1038/srep34653

**Published:** 2016-10-17

**Authors:** Elizabeth Thomas, Vidya Gopalakrishnan, Mahesh Hegde, Sujeet Kumar, Subhas S. Karki, Sathees C. Raghavan, Bibha Choudhary

**Affiliations:** 1 Department of Biochemistry, Indian Institute of Science, Bangalore 560 012, India; 2 Institute of Bioinformatics and Applied Biotechnology, Electronic City, Bangalore 560 100, India; 3 Department of Pharmaceutical Chemistry, KLE University’s College of Pharmacy, Bangalore 560 010, India

## Abstract

Resveratrol is one of the most widely studied bioactive plant polyphenols which possesses anticancer properties. Previously we have reported synthesis, characterization and identification of a novel resveratrol analog, SS28. In the present study, we show that SS28 induced cytotoxicity in several cancer cell lines *ex vivo* with an IC_50_ value of 3–5 μM. Mechanistic evaluation of effect of SS28 in non-small cell lung cancer cell line (A549) and T-cell leukemic cell line (CEM) showed that it inhibited Tubulin polymerization during cell division to cause cell cycle arrest at G2/M phase of the cell cycle at 12–18 h time period. Immunofluorescence studies confirmed the mitotic arrest upon treatment with SS28. Besides, we show that SS28 binds to Tubulin with a dissociation constant of 0.414 ± 0.11 μM. Further, SS28 treatment resulted in loss of mitochondrial membrane potential, activation of Caspase 9 and Caspase 3, leading to PARP-1 cleavage and finally cell death via intrinsic pathway of apoptosis. Importantly, treatment with SS28 resulted in regression of tumor in mice. Hence, our study reveals the antiproliferative activity of SS28 by disrupting microtubule dynamics by binding to its cellular target Tubulin and its potential to be developed as an anticancer molecule.

Microtubules are cytoskeletal filaments in cells composed of two proteins α and β Tubulin which are involved in many cellular functions such as formation of meiotic and mitotic spindle, cell motility, cell shape and transport of proteins and organelles^1^. Microtubules and their dynamics are targets of chemically diverse group of antimitotic compounds that are derived mostly from natural sources. Antimitotic agents inhibit cell proliferation by acting on the polymerization of spindle microtubules, which are essential for proper spindle function. Although the antimitotic drugs have been used clinically for treating diseases, the loss of efficacy over time due to development of resistance is considered as a major drawback^2^,^3^.

The most successful microtubule targeting drugs includes *Vinca* alkaloids (Vincristine, Vinblastine, Vinorelbine, Vindesine and Vinflunine), paclitaxel and docetaxel. Among these, Vincristine and Vinblastine are Tubulin binding agents, bind to the Vinca domain and inhibit Tubulin assembly^4^,^5^. In contrast, paclitaxel and docetaxel bind to the taxane-binding site and stabilize microtubules without increasing microtubule polymerisation^6^,^7^,^8^.

Resveratrol (trans-3,4′,5-trihydroxystilbene), a naturally occurring polyphenolic compound is highly enriched in the skin of red grapes, peanuts and various fruits. It has also been reported in the root of the plant, *Polygonum cuspidatum*, and an important constituent of Chinese and Japanese folk medicines^9^. Resveratrol has been extensively investigated as a cardioprotective, anti-inflammatory, and antiaging agent^10^,^11^,^12^. In addition, studies have shown that resveratrol has a strong chemopreventive effect against the development of cancers of skin, breast, prostate and lung^10^,^13^,^14^,^15^,^16^. Besides, effects of resveratrol in downregulation of angiogenesis-associated genes, activation of the apoptotic pathways and induction of cell cycle arrest have also been studied^17^,^18^,^19^,^20^.

Using resveratrol as the prototype, we have recently synthesised various analogs, evaluated their effect on cancer cell lines and identified (E)-1,2,3-trimethoxy-5-(4-methylstyryl)benzene (6 h) (SS28) as the potent molecule^21^. In the present study we evaluate antiproliferative activity of SS28 in different cancer cells and show that SS28 treatment leads to G2/M arrest. In addition, we have determined binding affinity of SS28 to purified Tubulin. Further, we show that SS28 interferes with Tubulin polymerization leading to disruption of the formation of microtubule, which in turn leads to metaphase arrest. Eventually, SS28 treatment results in cell death by activating the intrinsic pathway of apoptosis.

## Results

### SS28 induces cytotoxicity in different cancer cell lines

**Figure 1:**
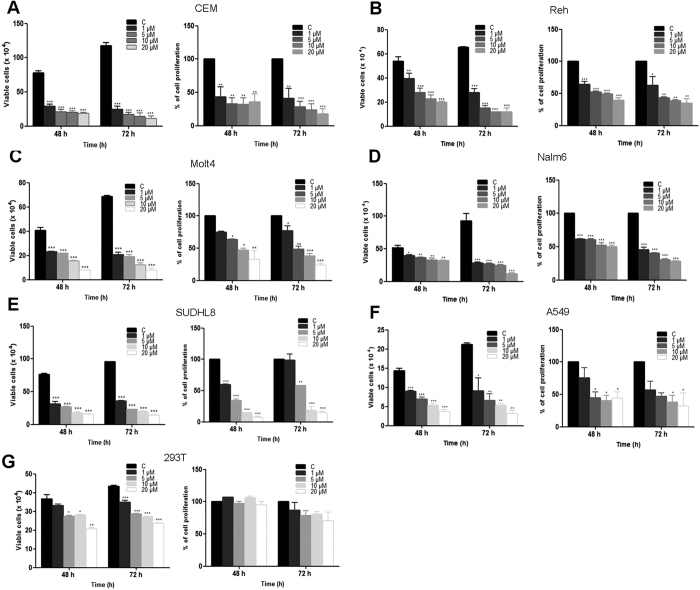
Effect of SS28 on cell viability in various cancer cell lines. Cells were cultured with increasing concentrations (1, 5, 10 and 20 μM) of SS28 for 48 and 72 h. Trypan blue and MTT assays were performed to assess the cytoxicity induced by SS28. Cells treated with DMSO served as vehicle control. Different cancer cell lines, CEM (**A**), Reh (**B**), Molt4 (**C**), Nalm6 (**D**), SUDHL8 (**E**), A549 (**F**) 293T (**G**) were used for the study. Each experiment was repeated a minimum of three times. Error bars indicate the SEM and P value was calculated by comparing the mean of control group with mean of SS28 treated group, *p < 0.05, **p < 0.005, ***p < 0.0001.

Various leukemic cell lines (CEM, Reh, Molt4 and Nalm6), human lung carcinoma cells (A549), cervical cancer cell line (HeLa), Diffuse large B cell lymphoma cell line (SUDHL8) and human embryonic kidney epithelial cell line (HEK293T) were used for evaluating their sensitivity towards SS28 (1, 5, 10 and 20 μM). Cells treated with DMSO were used as vehicle control. Results of trypan blue assays showed that SS28 interfered with cell viability in a dose dependent manner (Fig. 1). Among the cell lines tested CEM and A549 exhibited maximum sensitivity to SS28 followed by SUDHL8, Molt4 and Reh, whereas Nalm6 showed moderate sensitivity after 48 h of the treatment (Fig. 1). IC_50_ values of SS28 in CEM and A549 cell line were 2.6 and 5.2 μM, respectively, whereas in SUDHL8, Molt4, Reh and Nalm6 were 2.7, 5.1, 7.9 and 21 μM, respectively after 48 h of treatment (Suppl. Table 1). Among the cancer cell lines studied, HeLa cells exhibited least sensitivity to SS28 (32.4 μM) (Suppl. Table 1). Interestingly, SS28 induced only limited cytotoxicity on normal cell line HEK293T, compared to most of the cancer cells (Fig. 1). Besides, we also observed minimal effect on cell viability (48 h), when peripheral blood mononuclear cells (PBMC) were treated with SS28 with an IC_50_ value >35 μM (Suppl. Table 1). However, when mouse embryonic fibroblast cells (MEF) were evaluated for their sensitivity to SS28, it exhibited significant cell death with an IC_50_ value 5 μM (Suppl. Table 1). This is understandable as MEFs are known to proliferate efficiently and SS28 is most probably acting as a Tubulin inhibitor. Impact of SS28 treatment on cell proliferation was also evaluated using MTT assay. Results showed that the proliferation of cells was affected maximally in the case of CEM, A549, Molt4 and SUDHL8 which was followed by Reh and Nalm6 after 48 h of treatment (Fig. 1). Taken together, our results suggest that SS28 affected cancer cell growth in all leukemic cells and lung cancer cells tested.

### SS28 induces G2/M cell cycle arrest

**Figure 2:**
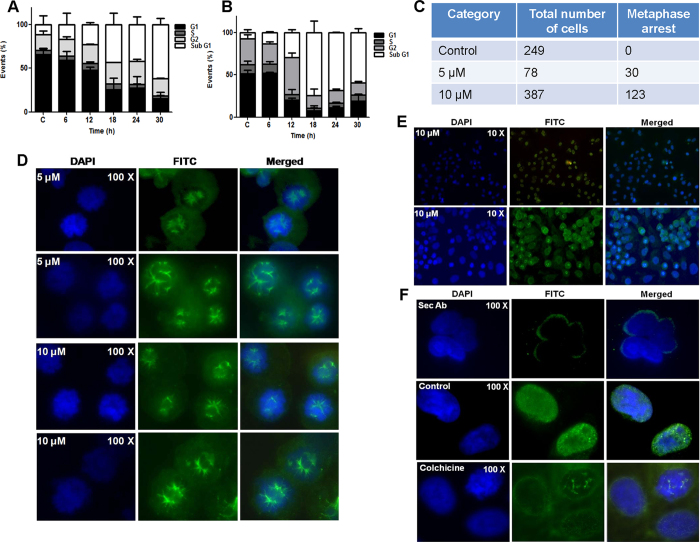
Effect of SS28 on cell cycle progression and spindle microtubule formation. (**A,B)** Bar diagram summarising the % of cells in each phase of cell cycle. A549 (**A**) and CEM (**B**) cells were treated with SS28, 5 and 2 μM, respectively for different time points (6, 12, 18, 24 and 30 h) and cell cycle phase distribution was analysed by flow cytometry. DMSO treated cells served as the vehicle control. **(C–F**) Effect of SS28 on spindle microtubule formation in A549 cells. A549 cells were cultured in presence of 5 and 10 μM of SS28 for 24 h. Cells were then fixed and labelled with a monoclonal α-Tubulin (green) antibody conjugated to FITC and nuclei stained with DAPI (blue) as described in materials and methods. (**C)** Table representing the metaphase arrest in the control and treated cells after 5 and 10 μM of SS28 treatment. Representative fluorescence images of control and treated cells in duplicates. (**D**) SS28 treated (5 and 10 μM) A549 cells showing bipolar spindles. (**E**) SS28 treated (10 μM) A549 cells (10X view). (**F**) Single cell view of secondary antibody control, primary antibody treated control cells and Colchicine treated cell. Images shown are DAPI, FITC and merged, respectively.

To examine whether the growth inhibition observed is due to cell cycle arrest followed by apoptosis, A549 and CEM cells were treated with SS28 (5 μM for A549 and 2 μM for CEM) for various time points (6, 12, 18, 24 and 30 h) and cell cycle distribution was studied by flow cytometry. Results showed significant accumulation of cells at G2/M phase in a time-dependent manner (6 to 24 h) and a subsequent increase in the sub-G1 population, indicative of apoptosis at 30 h in case of A549 (Fig. 2A; Suppl. Fig. 1A). Thus our result showed a prominent G2/M arrest following SS28 treatment in A549 cells.

Similarly, studies in CEM also showed a distinct G2/M arrest after 12 h of treatment, as compared to vehicle control (Fig. 2B; Suppl. Fig. 1B). However, further increase in incubation resulted in elevated levels of cell death accounting for observed increase in sub-G1 population. Therefore, our results suggest that SS28 treatment resulted in cell cycle arrest at G2/M phase leading to apoptosis upon further incubation.

### Effect of SS28 on mitotic spindle organization

Since we observed G2/M phase arrest during cell cycle analysis, we checked whether SS28 affects organisation of spindle microtubule during mitotic progression of cells. To investigate this, lung cancer cell line, A549 was chosen as it showed significant G2/M arrest. A549 cells treated with SS28 (0, 5 and 10 μM) for 24 h were processed for immunofluorescence microscopy studies (Fig. 2C). Results showed that ~38% of the cells displayed abnormal spindle organisation upon treatment with 5 μM of SS28 (Fig. 2C–F). Many cells had multipolar spindles, while others had aberrant bipolar spindles with misaligned chromosomes (Fig. 2D). Both multipolar spindles and abnormal bipolar spindles were also observed upon 10 μM SS28 treatment (Fig. 2C–F). The observed abnormal spindle organisation could be due to the binding of SS28 to Tubulin, thereby preventing microtubule polymerisation. A large fraction of cells also showed chromosome condensation which is a characteristic feature of prometaphase stage of mitosis (Fig. 2E).

Further we used Colchicine treated A549 cells as a positive control for the study (Fig. 2F). Colchicine is known to destabilise microtubule assembly by binding to Tubulin^19^,^22^. Upon treatment with Colchicine, expected chromosome condensation and metaphase arrest was observed as compared to untreated cells (Fig. 2F). Thus, the compound SS28 significantly affected the microtubule formation leading to chromosome condensation, which was comparable to that of Colchicine.

### SS28 affects the Tubulin polymerisation

**Figure 3:**
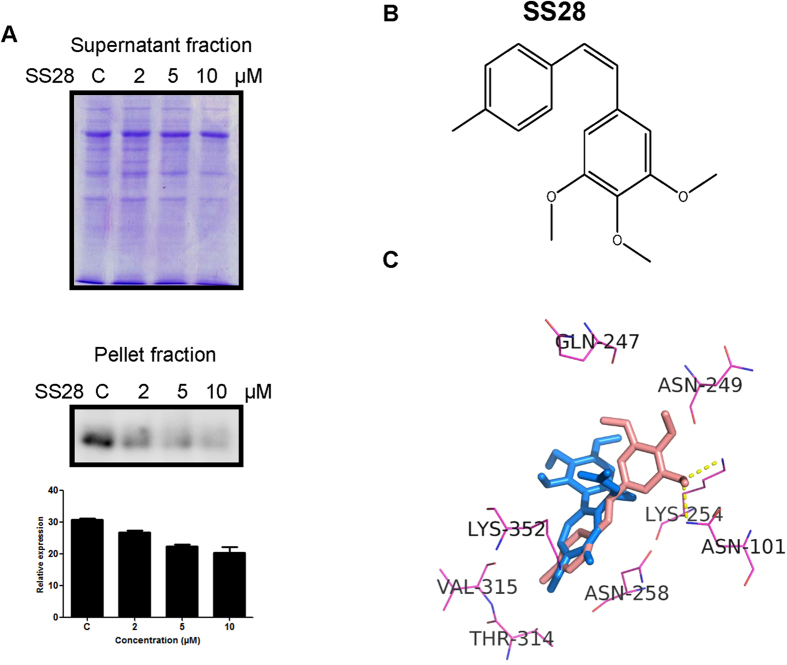
Docking studies showing SS28 interaction with Tubulin in colchicine binding site and the effect of SS28 on microtubule dynamics. (**A**) CEM cells were treated with SS28 (2, 5 and 10 μM) and processed for the microtubule assembly assay. Supernatant fractions were resolved in 8% SDS-PAGE. The pellet fractions were immunoblotted using α- Tubulin antibody and quantified. (**B**) Structure of SS28. (**C**) Docked pose of SS28 with Tubulin. SS28 can interact with the colchicine binding pocket of Tubulin with the hydrogen bond interaction with Lys 254 and Asn 101. SS28 represented in red and colchicine in blue colour.

Cell based Tubulin polymerisation assay was performed to check whether SS28 indeed affects the Tubulin polymerisation. Results showed that the pellet fraction containing the polymerised Tubulin decreased upon treatment with SS28 in a concentration dependent manner (Fig. 3A). In contrast, the supernatant fraction containing depolymerised microtubules remained equal in all the cases irrespective of the concentration (Fig. 3A). This suggests that SS28 inhibited the Tubulin polymerisation at a concentration of 2 μM onwards due to its binding to Tubulin.

To gain insights about SS28 interaction with Tubulin, docking studies were carried out using Autodock4.2.program. Results showed that, methyl benzene group of SS28 can fit in the methoxytropone ring (ring C) of colchicine binding site of Tubulin (Fig. 3B,C). Besides the trimethoxy benzene group of SS28 is involved in the hydrogen bond interactions with Lys 254 and Asn 101 of Tubulin chain with a favourable binding energy of −6.83 kcal/mol (Fig. 3C). Hence, docking studies revealed potential interaction of SS28 with Tubulin (Fig. 3C). Thus, our biochemical data in conjunction with docking studies suggest that SS28 can indeed target Tubulin within the cells.

### SS28 can bind to Tubulin

**Figure 4:**
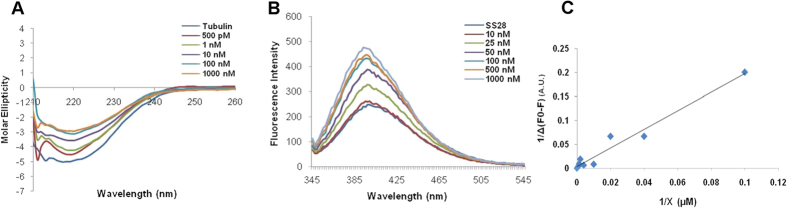
Evaluation of binding affinity of SS28 to Tubulin. **(A)** Circular dichroism studies were performed at 200–260 nm (4 °C) on a JASCO J-810 spectropolarimeter. The spectra of Tubulin alone (1 μM) in PEM buffer and in presence of increasing concentration of SS28 were recorded. Spectra of buffer containing corresponding concentrations of DMSO were subtracted from experimental data and presented. (**B)** Evaluation of change in fluorescence intensity when increasing concentration of Tubulin was added to SS28. Emission spectra of SS28 alone (10 μM) or with increasing concentration of Tubulin (10, 25, 50, 100, 500 and 1000 nM) were recorded following incubation at 30 °C for 30 min. The fluorescence intensities were plotted against the wavelength. (**C)** The double reciprocal plot of SS28 binding to Tubulin. X axis indicates the concentration of Tubulin and Y axis represents the differences in the fluorescence intensity.

To test whether SS28 can directly bind to purified Tubulin, purified protein was incubated with increasing concentration of ligand (500 pM, 1, 10, 100, 1000 nM) in PEM buffer and circular dichroism (CD) was performed. Results showed a shift in the spectrum when SS28 was added from as low as 500 pM (Fig. 4A). Thus, addition of SS28 to Tubulin significantly increased the molar ellipticity suggesting alteration in the secondary structure of Tubulin. Further, fluorescence studies were conducted to evaluate the binding affinity of SS28 to Tubulin. SS28 exhibited fluorescence in aqueous solution, with an emission maximum at 399 nm. The binding to purified Tubulin results in an induction of fluorescence in a concentration dependent manner (Fig. 4B). The change in fluorescence of SS28 was used to determine both association and dissociation. The double reciprocal plot of fluorescence data yielded a dissociation constant (Kd) of 0.414 ± 0.11 μM with a binding ratio 1:1 (Fig. 4C).

### SS28 treatment did not result in ROS production

Intracellular ROS production was tested following treatment with SS28 (2 μM) in CEM cells by flow cytometry after staining with H_2_DCFDA (Suppl. Fig. 2A). Results did not show generation of ROS when tested for an incubation period as early as 5 min or at later time points (Suppl. Fig. 2). In contrast, the cells treated with H_2_O_2_ showed a distinct ROS production which served as positive control (Suppl. Fig. 2). Hence, our results suggest that SS28 treatment does not result in ROS production indicating that cell death is not caused by oxidative stress.

### SS28 treatment affected the mitochondrial membrane potential

**Figure 5:**
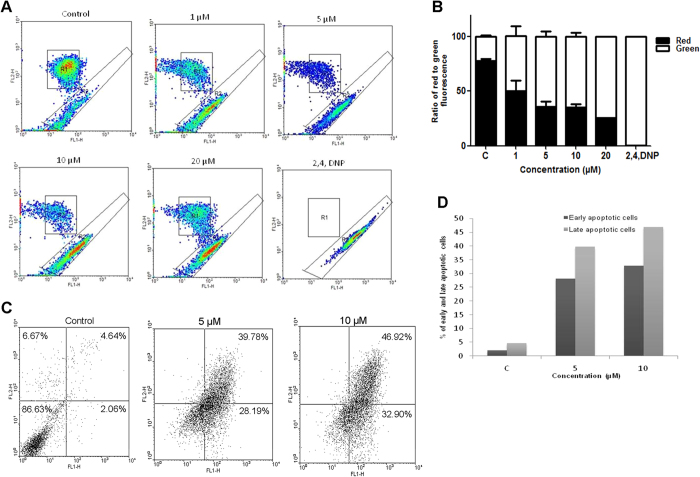
Effect of SS28 treatment on mitochondrial membrane potential and induction of apoptosis. CEM cells were treated with SS28 (1, 5, 10, 20 μM) for 48 h, harvested and stained with JC-1 dye and analyzed by flow cytometry. (**A**) Upper region represents the healthy cells that emit red fluorescence whereas the lower region emits the green fluorescence showing the apoptotic population. 2,4 Dinitrophenol was used as the positive control. (**B)** Bar graphs represent the ratio of red to green fluorescence. (**C**,**D)** Annexin V-FITC and PI staining to evaluate the effect of SS28 on CEM cells. CEM cells were treated with SS28 and % of apoptotic cells was determined by staining with Annexin V-FITC and PI. In each panel, the lower left quadrant shows cells which are negative for both PI and Annexin V-FITC, upper left shows only PI cells which are necrotic. The lower right quadrant shows Annexin positive cells which are in the early apoptotic stage and the upper right shows the both Annexin/PI positive, which are in the late apoptosis necrosis. (**C**) Control cells and cells treated with SS28 are shown. (**D)** Histogram represents the percentage of early and late apoptotic cells.

Mitochondrial membrane potential plays an important role in triggering apoptosis by regulating the membrane permeability^23^. Therefore, we checked the effect of SS28 treatment on mitochondrial membrane potential in CEM cells using JC-1 staining followed by flow cytometry (Fig. 5A). The JC-1 dye, due to an inherent positive charge enters the mitochondrial matrix due to the negative charge established by the intact mitochondrial membrane potential. When intact mitochondrial membrane potential is absent due to a chemical treatment, JC-1 accumulates in the cytoplasm in monomeric form characterized by green fluorescence. Upon treatment with SS28, a concentration dependant increase in the green fluoresced cells was observed in contrast to control cells which exhibited maximum red fluorescence (Fig. 5A,B). Hence our results suggest the loss of mitochondrial membrane potential upon treatment with SS28.

### Treatment with SS28 resulted in apoptosis rather than necrosis

Previous studies suggested that microtubule-inhibiting agents cause G2/M phase arrest followed by apoptosis^24^,^25^. Hence, we used Annexin V-FITC/PI double-staining method to study apoptosis and necrosis following exposure to SS28 (5 and 10 μM for 48 h) in CEM cells. Results showed that SS28 induced apoptosis in a concentration dependent manner (Fig. 5C). At 5 μM, 39.8% of cells were in late apoptosis while 28.2% in early apoptotic stage, whereas they were 46.9% and 32.9% respectively, when treated with 10 μM of SS28 (Fig. 5C,D). However, we did not observe any cells undergoing necrosis (Fig. 5C).

### SS28 treatment lead to alteration in the expression levels of cell cycle and apoptotic proteins

**Figure 6:**
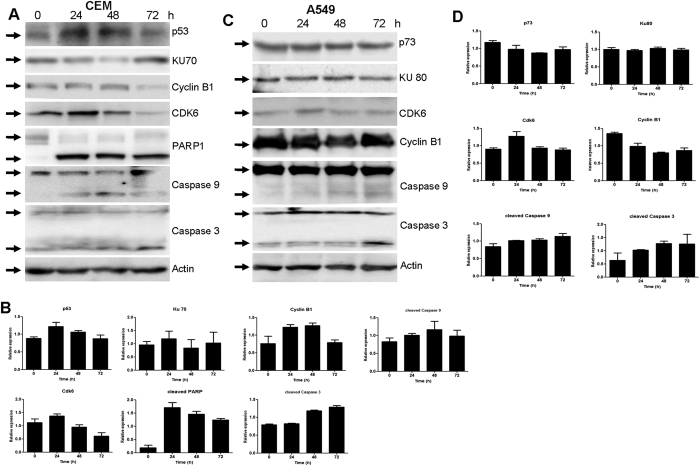
Expression levels of apoptotic proteins and cell cycle regulatory proteins in A549 and CEM cell lines upon treatment with SS28. A549 and CEM cells were treated with SS28 (5 μM) for 24, 48 and 72 h. Whole cell lysate was prepared and proteins were resolved on a SDS-PAGE and western blotting was performed using specific primary and secondary antibodies. Blots shown are representative blots of two independent experiments with identical results. Actin was used as the loading control. (**A**) For CEM cells p53, Ku70, Cyclin B1, Cdk6, PARP-1, Caspase 9 and Caspase 3 proteins were evaluated. (**B**) Quantification of the proteins shown in panel A is represented as bar diagram with error bars. (**C**) For A549 cells, p73, Ku80, Cdk6, Cyclin B1, Caspase 9 and Caspase 3 proteins were evaluated. (**D**) Quantification of the respective proteins is shown in bar diagram with error bars.

In order to study the mechanism by which SS28 induces cell cycle arrest and apoptosis, we studied the expression levels of some of the cell cycle and apoptotic markers. To accomplish this, CEM and A549 cells were treated with SS28 (5 μM for 24, 48 and 72 h), cell lysates were prepared and used for western blotting studies. The level of Cyclin B1, a key regulator of cells entry into mitosis, was increased at 48 h time point in CEM cells followed by a decrease at 72 h (Fig. 6A,B). However, in case of A549 cell line, the Cyclin B1 level was maintained throughout the time period, but with a moderate decrease at 48 h (Fig. 6C,D). The expression level of Cdk6 was increased at 24 h followed by a decrease at 48 and 72 h in both the cell lines tested (Fig. 6).

To further investigate the underlying mechanism of action of SS28 on cancer cell lines, we examined the possible involvement of apoptotic proteins. Caspase 9, an indicator of intrinsic pathway of apoptosis, showed an increase in the cleaved Caspase 9 level (Fig. 6). Another marker of apoptosis, Caspase 3 also showed activation in both the cancer cells, further suggesting activation of apoptosis (Fig. 6). Besides, we also observed activation of PARP1 upon treatment with SS28 in CEM cells. Since activation of apoptosis was dependent on Caspase 3, we checked the expression levels of p53. Results showed an upregulation of p53 at 24 h time point followed by a decrease in the later time points (Fig. 6A,B). However, in A549 cells the expression levels of p73, which is also involved in the induction of apoptosis remained same (Fig. 6C,D).

### SS28 showed inhibition of tumor progression in mice

**Figure 7:**
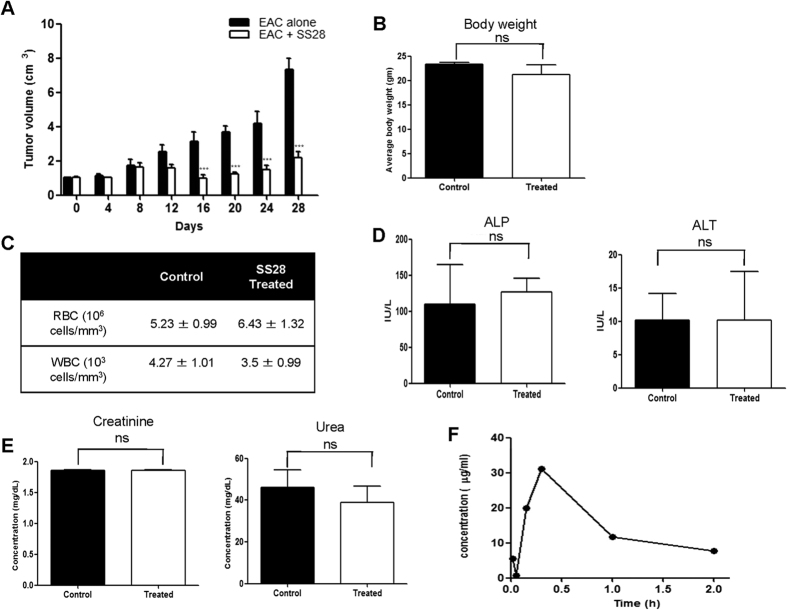
Impact of SS28 on tumor bearing mouse and evaluation of side effects of SS28 in normal BALB/c mice. (**A**) Nine doses of SS28 (15 mg/kg. b.wt.) were administered orally on every alternate day from 12^th^ day of EAC cell injection in mice. Data shows volume of tumor measured at different time intervals, with and without treatment of SS28. Results depicted from three independent batches of experiments containing 10 animals each. (**B**) Mice were orally administered with nine doses of SS28 (15 mg/kg) on every alternate day. Data represented as average body weight in both the controls and SS28 treated mice. Error bars indicate SEM. (**C–E)** Hematological profile and renal and liver functional assay. Blood was collected on the 21^st^ day of the treatment counted RBC and WBC (**C**) and serum was tested for alkaline phosphatase (ALP), alanine aminotransferase (ALT) (**D**), urea and creatinine (**E**). Columns represent mean from five animals in each group; bars SEM. (**F**) Pharmacokinetics of SS28 in mice plasma. SS28 was injected intraperitonealy to mice and blood was collected and processed as described in materials and methods. Data represented in time points versus the peak area as the function of the concentration of SS28 in plasma.

In order to examine the *in vivo* effect of SS28 on tumor cell death in a mouse model, tumor was induced in BALB/c mice using EAC cells. A total of 10 mice were used per batch which included 5 tumor bearing mice (control) and 5 tumor mice treated with SS28 (experimental). The experiment was repeated three independent times. Based on the preliminary studies, we selected a dose of 15 mg/kg body weight for the investigation. After 12^th^ day of EAC injection, when small sized tumor was visible, the animals were treated with nine doses of SS28 (every alternate day). Results showed that there was no further tumor progression in the mice when SS28 was administered, unlike the untreated tumor control mice (Fig. 7A). Tumor progression was analysed upto 28 days in the study. Therefore, we observed that SS28 treatment resulted in inhibition of tumor cell proliferation, although we did not observe complete regression of the tumor.

### Effect of SS28 on normal mice

In order to evaluate possible side effects due to SS28 treatment, a total of 10 BALB/c mice were used. Among them five animals were administered with SS28 (15 mg/kg, 9 doses) while other 5 mice were used as tumor control without any treatment. Results showed that there was no significant difference in body weight of the animals following SS28 treatment (Fig. 7B). To further check the side effects, we performed haematological, liver and kidney function assays. The mice were sacrificed on 21^st^ day of the treatment and used for the investigation. Results showed no significant difference in the number of RBCs and WBCs upon treatment with SS28 (Fig. 7C). Further, we found that the ALP level was slightly higher in the treated animals. But there was no significant difference in the level of ALT, the marker for normal liver function, upon treatment with SS28 (Fig. 7D). Further, there was no significant difference in the levels of creatinine and urea in the serum which indicates efficient kidney function (Fig. 7E). Thus, our study indicated that SS28 treatment did not result in any major toxicity on functions of normal tissues.

### Pharmacokinetics of SS28 in mice models

SS28 was administered intraperitonealy and the concentration of the compound was measured in plasma using a standard plot. Blood was drawn from the heart at 15 min, 30 min, 1 and 2 h of the administration of SS28. Bioavailability data were obtained by measuring the concentration by high-performance liquid chromatography in serum samples taken at different times after SS28 administration (Fig. 7F). Results showed a significant increase in the level of SS28 in the plasma when collected 30 min after the injection, although the compound was detectable both at 15 min and 1 h post-treatment (Fig. 7F). We observed a significant decrease in the levels of SS28 after 1 h of treatment.

## Discussion

Resveratrol, *trans*-3,5,4′-trihydroxy-*trans*-stilbene, is a natural polyphenol with potent chemopreventive and chemotherapeutic properties^9^,^26^. It can modulate multiple cellular processes, including apoptosis, cell cycle progression, inflammation and angiogenesis^26^. Moreover, previous studies have shown that hydroxy stilbenes and its derivatives can act as potential anticancer agents by targeting microtubule assembly dynamics^27^,^28^. In view of the cancer preventive and chemotherapeutic property of resveratrol, we synthesised and characterised a series of resveratrol derivatives and identified SS28 as a potent anticancer molecule^21^.

In the present study, we have investigated the mechanism of action of SS28 with its molecular target, Tubulin by binding studies in conjunction with the cell based assays, *ex vivo* and *in vivo*. We show that SS28 induced cytotoxicity in most of the cancer cell lines investigated, in a concentration-dependent manner. SS28 showed only limited cytotoxicty on kidney epithelial cell line and PBMCs. SS28 treatment led to G2/M phase arrest in both A549 and CEM cells followed by increased accumulation of cells in Sub G1 phase in a time dependent manner suggesting activation of cell death pathways following induction of cell cycle arrest. Previous studies have shown such a cell cycle arrest by various chemotherapeutic agents. Examples include nocodazole and paclitaxel, both targeting microtubule formation resulting in the G2/M cell cycle arrest followed by apoptosis both *ex vivo*^29^ and *in vivo*^30^. Besides, the observed regulation of expression of checkpoint proteins corresponding to the cell cycle dynamics can also explain the cell cycle arrest mediated by SS28. Cyclin B1 is known to be involved in G2/M cell cycle progression, and is a regulator for G2/M transition^31^. SS28 treatment led to decrease in the cyclin B1 level further explaining the observed cell cycle arrest.

The assembly dynamics of spindle microtubules is crucial for chromosome segregation during mitosis. It has been shown that many antimitotic drugs inhibit mitosis at the metaphase/anaphase transition at lower concentrations primarily by suppressing microtubule dynamics^32^,^33^,^34^,^35^,^36^. We find that SS28 induced disassembly of microtubules with misconfigured chromosomes in A549 cell line. The observed effect is comparable to many other potent antimitotic drugs with respect to morphology of spindles in blocked cells^1^,^37^,^38^. Interestingly, we noted that SS28 treated cells exhibited more distinct structures than the colchicine treated positive controls suggesting that SS28 has strong affinity to interact with Tubulin in cells, thereby inhibiting the microtubule assembly compared to colchicine. Furthermore, the microtubule assembly assay suggests that SS28 inhibits the Tubulin polymerisation by binding to Tubulin in a concentration dependent manner.

Docking studies indicated that SS28 can bind to colchicine binding pocket in Tubulin. CD studies revealed that SS28 can bind to Tubulin leading to increased molar ellipticity indicating conformational change in protein following SS28 binding (Fig. 4A). Besides, fluorescence studies suggested a dissociation constant of 0.414 ± 0.11 μM indicating that the binding affinity of SS28 to Tubulin is comparable to that of colchicine^39^. Moreover, SS28 exhibited an increase in fluorescence upon binding to Tubulin, which was comparable to that known for colchicine^40^. The molecular modeling data showed that methyl benzene group of SS28 can fit in the methoxytropone ring (ring C) of colchicine binding site on Tubulin (Fig. 3C). The results of docking studies were further supported by preliminary competition studies (data not shown). The change in the fluorescence spectra upon addition of SS28 and colchicine together indicate the binding of SS28 to the colchicine binding site on Tubulin. In support of this data, AC, a colchicine analog, which has A and C ring, which binds to Tubulin rapidly and reversibly to its colchicine site shows increase of fluorescence^41^.

In an earlier study, it has been shown that methylated derivatives of flavonoids exhibit higher antiproliferative potency on cancer cells than their hydroxylated counterparts^42^. In the present study, the small molecule (SS28) evaluated is a methoxy derivative of resveratrol exhibiting significant anticancer properties by affecting Tubulin proliferation. Increased lipophilic properties of the methoxy derivative of resveratrol can help in increased uptake through the cell membrane, besides targeting Tubulin. Our results also support the notion that methylation of the hydroxyl groups, could be the critical modification required for interference of Tubulin polymerization.

Annexin V FITC/PI staining showed presence of both early and late apoptotic cells, which increased in a dose-dependent manner upon treatment with SS28. However, we did not find cells undergoing necrosis, which was also consistent with previous studies using resveratrol based inhibitors. Thus, cytotoxicity induced by SS28 was mainly due to apoptosis rather than necrosis. Further, the observed reduction in the mitochondrial membrane potential following treatment is consistent with induction of apoptosis by SS28. Caspase family plays a key role in apoptosis through the proteolysis of specific targets. Intracellular caspases are activated during apoptosis via two major pathways, mitochondria-mediated pathway and death receptor-mediated pathway^43^. Expression analysis showed activation of Caspase 3, Caspase 9 and PARP-1 suggesting activation of intrinsic pathway of apoptosis^23^,^43^,^44^.

Our data also showed that SS28 inhibited tumor cell proliferation in mouse bearing EAC tumor. EAC cells possessing malignant features of cancer are commonly used for inducing tumors in mice and for evaluating anticancer activity of small molecules *in vivo*^45^,^46^. Our results showed that in control animals, the tumor showed unrestricted progression, whereas in SS28 treated mice, a significant reduction in the tumor size was observed. Hematological and enzymatic assays showed that SS28 did not interfere with other cellular functions. Thus, as a proof of principle we show that the novel Tubulin inhibitor identified here inhibits tumor progression in mice without major side effects.

## Conclusion

In summary, the results of the present study show that SS28, a resveratrol based Tubulin inhibitor can function as an antimitotic agent and inhibit cell proliferation in various cancer cell lines. SS28 acts by disrupting the microtubule formation by binding to Tubulin leading to cell death, which is mediated through intrinsic pathway of apoptosis in cell lines and mouse tumor tissues. Thus, our results suggest that SS28 can act as a potent microtubule targeting agent and further can be developed as an anticancer drug.

## Methods

### Chemicals and reagents

All the chemicals and reagents used in the present study were obtained from Sigma Chemical Co (St. Louis, MO) and SRL (India). Antibodies were purchased from Santa Cruz Biotechnology (USA) and Cell Signaling Technology (USA). Purified Tubulin was purchased from MP Biomedicals (France). Synthesis and characterization of SS28 [(E)-1,2,3-trimethoxy-5-(4-methylstyryl)benzene (6 h)] has been described previously^21^.

### Cell lines and culture conditions

Human T-cell leukemic cell line, CEM; human lung carcinoma cell line, A549; Human T-cell leukemic cell line, Molt4; human cervix adenocarcinoma cell line, HeLa; human embryonic kidney epithelial cell line, 293T, Mouse embryonic fibroblast (MEF) were purchased from National Centre for Cell Science, Pune, India. Human B cell leukemic cell lines, Reh and Nalm6 were a kind gift from Dr. M. Lieber and Diffuse large B cell lymphoma cell line, SUDHL8 was a gift from Dr. A. Epstein, USA and PBMC purchased from Saarum Biosciences, India. Cells were cultured in RPMI/MEM/Ham’s F12 media 1640 (Sera Lab, USA) containing 10% FBS (GIBCO BRL, USA), 100 U of Penicillin G/ml and 100 mg/ml of streptomycin/ml at 37 °C in a humidified atmosphere containing 5% CO_2_.

### Molecular modeling of SS28-Tubulin complex

3D structure of SS28 was generated in Discovery studio package using in-built dreiding-like force field (Discovery Studio 4.0. Accelrys, USA). 3D crystal structure of α, β-Tubulin bound with colchicine was retrieved from protein databank (PDB ID: 3UT5, www.rcsb.org/pdb)^17^. Molecular docking studies were carried out with Autodock4.2 program^47^. Briefly, SS28 was docked in colchicine binding site keeping the grid centres 9.45, 56.62 and 87.95 for X, Y and Z respectively (npts = 50 for X, Y and Z, spacing = 0.375). Rigid docking was carried out using Lamarckian genetic algorithm (GA run was kept 20 with medium search parameter). Best pose was selected according to lower binding energy and presented in the final image using Pymol.

### Circular dichroism

Purified Tubulin (1 μM) was resuspended in PEM buffer (80 mM PIPES (pH 6.9), 2 mM MgCl_2_, 0.5 mM EGTA, 1.0 mM GTP and 5% glycerol) and CD spectrum was recorded at a wavelength of 200 to 260 nm (4 °C) on a JASCO J-810 spectropolarimeter. 3 cycles were acquired for each sample at a scan speed of 100 nm/sec. Increasing concentration of SS28 (500 pM, 1, 10, 100 and 1000 nM) was incubated with Tubulin (1 μM) in PEM buffer and the spectrum was recorded. Spectral measurements were taken for buffer alone and buffer containing DMSO were subtracted from experimental data. The molar ellipticity was calculated using Spectra Manager software and plotted as a function of wavelength.

### Fluorescence polarisation

Fluorescence spectral measurements were performed using Infinite M200 PRO microplate reader (Tecan Group Ltd., Switzerland). Briefly, SS28 (10 μM) was preincubated with increasing concentration of Tubulin (10, 25, 50, 100, 500 and 1000 nM) at 30 °C for 30 min. SS28 Tubulin complex was then scanned for the emission spectra from 345 to 545 nm at 315 nm excitation range. Resulting fluorescence intensity was plotted as the function of wavelength.

The dissociation constant (Kd) was determined using double reciprocal plot of change in fluorescence of SS28 (1/Δ(F0-F)) as a function of protein concentration by the method of Benesi and Hildebrand assuming a 1:1 binding of SS28 to Tubulin^48^. The spectral measurements were recorded three times and SEM was calculated.

### Cell proliferation assays

The effect of SS28 on viability of CEM, Reh, Nalm6, SUDHL8, Molt4, A549, HeLa 293T, mouse embryonic fibroblast (MEF) and PBMC was determined by trypan blue dye exclusion assay as described previously^49^,^50^. Briefly, cells (0.75 × 10^5^ cells/ml) were treated with increasing concentrations of SS28 (1, 5, 10 and 20, μM) for 48 and 72 h. DMSO treated cells were used as the vehicle control. Cells were harvested and subjected to trypan blue assay. Data from a minimum of three independent experiments is presented as a histogram with error bars.

MTT (3-(4,5-dimethylthiazol-2-yl)-2,5-diphenyltetrazoliumbromide) assay was performed to determine the effect of SS28 on proliferation of cells as described^51^,^52^. Briefly, CEM, Reh, Nalm6, SUDHL8, Molt4, A549, HeLa and 293T cells (0.5 × 10^5^ cells/ml) were seeded in 24 well plate and treated with increasing concentrations of SS28 (1, 5, 10, 20 μM). MTT assay was performed after 48 and 72 h of incubation. The absorbance of experimental samples were divided by absorbance of untreated control, and presented as % of inhibition and shown as a bar diagram. DMSO treated cells, served as vehicle control. Error bars were calculated based on a minimum of three independent experiments and data is presented as histogram.

### Cell cycle phase distribution

Cell cycle analysis by flow cytometry was performed as previously described^53^. Briefly, 75,000 cells/ml (A549) were seeded in 6 well plates and grown in serum free medium for 18 h. After synchronisation, the cells were treated with 5 μM of SS28 (6, 12, 18, 24 and 30 h) and cells were processed as described before^53^,^54^. Similarly, CEM cells (75,000 cells/ml) were treated with SS28 (2 μM) and harvested (6, 12, 18, 24 and 30 h) and processed. Following fixation, cells were resuspended in PBS and stained with propidium iodide and evaluated in a flow cytometer (FACS Calibur, Becton Dickinson) using Cell Quest Pro software using excitation 488 nm laser and emission at 560/570 nm. A minimum of 10,000 cells were acquired per sample and histograms were analysed using WinMDI 2.8 software.

### Annexin V-FITC/PI double-staining assay

When cells undergo apoptosis, the integrity of the cell membrane is disrupted and phosphatidyl serine is exposed. Annexin V conjugated with FITC (apoptosis detection kit Santacruz, USA) has a selective affinity for phosphatidyl serine, based on which early and late apoptotic cells can be detected.

CEM cells (1 × 10^5^ cells/ml) were treated with SS28 (5 and 10 μM) for 48 h. Cells were then harvested, washed with cold PBS and used for annexin-PI double-staining assay^55^,^56^. Samples were resuspended in 1x binding buffer and incubated with Annexin V-FITC (0.4 g/μl) and PI (0.05 mg/ml) and analyzed by flow cytometry (FACS calibur) using CellQuest pro software at an excitation with 488 nm laser and emission at 530 nm^55^. A minimum of 10,000 cells were acquired for each sample and illustrated as dot plot using Flowing software.

### Determination of mitochondrial transmembrane potential

Flow cytometric analysis of cells stained with JC-1 was used to measure changes in mitochondrial transmembrane potential as described^49^,^56^. Briefly, cells were seeded at a density of 1 × 10^5^ cells/ml and treated with SS28 (1, 5, 10 and 20 μM). After 48 h, cells were harvested, washed and incubated with JC-1 (5,5′,6,6 tetrachloro-1,1′,3,3′-tetraethyl benzimidazol carbocyanine iodide; Calbiochem, USA) for 15 min. 2,4-Dinitrophenol-treated cells (2,4-DNP) served as the positive control. Cells were processed and analyzed by flow cytometry using CellQuest pro software with an excitation at 488 nm laser and emission at 530 nm. JC-1 monomers emit at 530 nm and J-aggregates emit at 590 nm.

### Assay for intracellular ROS production

Intracellular ROS generation in cells was assessed using the oxidation-sensitive fluorescent probe 2,7-dichlorodihydro fluorescein diacetate (H_2_DCFDA) as previously described^52^,^57^. CEM cells were treated with SS28 (5 μM) for 5, 10, 15, 30 and 60 min, harvested, washed and incubated with H_2_DCFDA (30 min at 37 °C) and then fluorescence intensity was analyzed by flow cytometry. H_2_O_2_ was used as the positive control.

### Immunofluorescence microscopy

Immunofluorescence microscopy was performed as previously described^51^,^58^. A549 cells were grown on coverslips coated with gelatin (0.1%). Cells were treated with SS28 (5 μM, 24 h) and were fixed with 2% p-formaldehyde (20 min at RT). After 24 h of the treatment nonspecific antibody binding sites were blocked using FBS following which cells were incubated with antitubulin antibody (1:500) at RT. Coverslips were washed in PBS, incubated in biotinylated antimouse secondary antibody (1:500; RT for 1 h) followed by Streptavidin-FITC (1:200). Finally, cells were mounted with DABCO and images were captured using Zeiss Fluorescence microscope and analyzed using Axio vision software. Colchicine treated cells were used as a positive control.

### Microtubule assembly assay

Separation of insoluble polymerized Tubulin from soluble Tubulin dimers was performed as described^59^. In brief, CEM cells (1 × 10^6^) were treated with 2, 5 and 10 μM of SS28 for 24 h. Cells were harvested and washed with PBS before adding polymerization lysate buffer containing 20 mM Tris-HCl (pH 6.8), 1 mM MgCl_2_, 2 mM EGTA, 20 g/ml aprotinin, 20 g/ml leupeptin, 1 mM phenylmethylsulfonyl fluoride, 1 mM orthovanadate and 0.5% NP-40. After centrifugation of the lysates at 15,000 × g for 10 min at 4 °C, the pellets were resuspended in SDS-PAGE loading buffer and dissolved by heating at 95 °C for 10 min. α-Tubulin was then immunoblotted for the pellet fraction containing polymerized microtubules and supernatants containing depolymerised microtubules were equalised and resolved on 8% SDS PAGE.

### Immunoblotting

Cell lysate was prepared after treating with SS28 (5 μM for 24, 48 and 72 h) from CEM and A549 cell lines^58^,^60^. In brief, cells were harvested, washed with PBS, resuspended in lysis buffer (RIPA, 25 mM Tris (pH 7.6), 150 mM NaCl, 1% NP-40, 1% sodium deoxycholate and 0.1% SDS) containing protease inhibitors. The protein lysate was cleared by centrifugation (14,000 rpm, for 15 min). The protein concentration in the supernatant was determined using the Bradford’s assay.

For western blot analysis, ~40 μg proteins were resolved over 8–10% SDS–polyacrylamide gel^60^,^61^,^62^. Following gel electrophoresis, proteins were transferred to PVDF membrane (Millipore, USAQ9). The primary antibodies used were against Caspase 3, Caspase 9, p73, p53, KU70, KU80, Cyclin B1, CDK6, PARP1 and Actin (Santa Cruz Biotechnology, USA). The membrane was incubated with appropriate HRP-conjugated secondary antibody (2 h at 4° C). The blots were developed using chemiluminescent solution (Immobilon^TM^ western, Millipore) and scanned by gel documentation system (LAS 3000, FUJI, Japan). Blots were stripped subsequently as per standard protocols and reprobed with anti β-Actin antibody.

### *In vivo* experiments

#### Animals

All the animal experiments were performed as per the principles and guidelines of the ethical committee for animal care of Indian Institute of Science (IISc) in accordance with Indian National Law on animal care and use. The Institutional Animal Ethics Committee of Indian Institute of Science, Bangalore, India approved the experimental design of the present study (Ref. CAF/Ethics/288/2012). BALB/c mice, 8–10 week old, weighing 18–22 g were purchased from central animal facility, IISc, India and used for the study. The animals were housed in polypropylene cages and provided standard pellet diet (Agro Corporation Pvt. Ltd., Bangalore, India) and water ad libitum. The standard pellet diet is composed of 21% protein, 5% lipids, 4% crude fiber, 8% ash, 1% calcium, 0.6% phosphorus, 3.4% glucose, 2% vitamin, and 55% nitrogen-free extract (carbohydrates). The mice were maintained under controlled conditions of temperature and humidity with a 12 h light/dark cycle.

#### Preparation of Ehrlich ascites carcinoma (EAC) cells for induction of tumor

EAC cells were collected from donor mice (Swiss albino) and resuspended in sterile saline. A fixed number of viable cells were injected into the peritoneal cavity of each recipient mouse and were allowed to multiply. The cells were withdrawn, diluted in saline and injected (15 × 10^5^ cells/animal) into the right thigh tissue of experimental animals for developing solid tumor.

#### Evaluation of antitumor activity of SS28 in mice models

A total number of 30 BALB/c mice were divided into three batches and were used for the present study. EAC cells (15 × 10^5^ cells/animal) were injected into right thigh for the development of solid tumor as described previously^57^,^61^,^63^. Among the 10 animals in each batch, 5 animals served as tumor control and the rest 5 animals received SS28 treatment (15 mg/kg b.wt.) by oral administration using gastric gavage. The dose used was selected based on preliminary studies. The first dose was given after 12^th^ day of tumor development (9 doses over a period of 3 weeks). The diameter of developing tumor was measured using vernier calipers at alternative days for entire life span of tumor animal and tumor volume was calculated using the formula V = 0.5 × a × b^2^, where “a” and “b” indicates the major and minor diameter, respectively^61^.

#### Evaluation of toxicity of SS28 in normal mice

A total number of 10 BALB/c mice were used for the study, of which 5 animals served as normal control while, remaining 5 were treated with SS28 (15 mg/kg). Body weight of each animal was monitored throughout the experiment and average body weight was calculated on 21^st^ day of treatment. In order to evaluate the effect of SS28 on physiological functions, blood was collected (21^st^ day) and analysed as described earlier^61^. The blood plasma was analyzed using Neubauer’s chamber, and mean values of total red blood cells (RBCs) and white blood cells (WBCs) were determined. Serum was separated and kidney and liver function tests were performed for each animal, to determine alkaline phosphatase (ALP), alkaline transferase (ALT), creatinine and urea levels as described earlier^61^. Values are presented as mean ± SEM for both the controls and SS28 administered mice.

### *In vivo* pharmacokinetics study

#### HPLC analysis of serum

Control and SS28 (15 mg/kg b.wt.) treated BALB/c mice were sacrificed and blood was collected at various time points (15, 30 min, 1 and 2 h; 2 each/time point) as described^9^,^26^. Plasma (50 μl) was then extracted with acetonitrile, centrifuged (3,000 × g for 15 min) to remove precipitated proteins and the resulting supernatants were analyzed using HPLC (Waters, USA).

Standard solution of SS28 was prepared by dissolving increasing concentration (25 to 100 μM) of the compound in acetonitrile. Standard calibration curves were plotted using peak area against the concentration of the compound and the retention time of the compound was also determined. Plasma concentrations of the treated groups were extrapolated from standard curves constructed by linear regression in Graph-Pad Prism 4.0 software (GraphPad Software, Inc.).

#### Statistical Analysis

As specified, values in tables and figures are expressed as the mean ± SEM of two to three independent experiments. Statistical comparisons were made by one-way ANOVA followed by Student’s t test using Graph-Pad Prism 4.0 software (GraphPad Software, Inc.). A probability value of <0.05 was considered to be significant.

## Additional Information

**How to cite this article**: Thomas, E. *et al*. A Novel Resveratrol Based Tubulin Inhibitor Induces Mitotic Arrest and Activates Apoptosis in Cancer Cells. *Sci. Rep.* **6**, 34653; doi: 10.1038/srep34653 (2016).

## Supplementary Material

Supplementary Information
