# Supplementary material for: A Novel Resveratrol Based Tubulin Inhibitor Induces Mitotic Arrest and Activates Apoptosis in Cancer Cells
Source: Sci Rep. 2016 Oct 17;6:34653. doi: 10.1038/srep34653 (PMC5066224; doi:10.1038/srep34653)
Supplement: Supplementary Information [file srep34653-s1.pdf]

# A Novel Resveratrol Based Tubulin Inhibitor Induces Mitotic Arrest and Activates Apoptosis in Cancer Cells


Elizabeth Thomas[1], Vidya Gopalakrishnan[2], Mahesh Hegde[1], Sujeet Kumar[3], Subhas S. Karki[3], Sathees C. Raghavan[1*] and Bibha Choudhary[2*]

[1]Department of Biochemistry, Indian Institute of Science, Bangalore 560 012, India, [2]Institute of Bioinformatics and Applied Biotechnology, Electronic City, Bangalore 560 100, India
[3]Department of Pharmaceutical Chemistry, KLE University's College of Pharmacy, Bangalore 560 010, India.

Correspondance

*Sathees C. Raghavan Phone : 091 80 2293 2674; Fax: 080 2360 0814

e-mail : sathees@biochem.iisc.ernet.in

*Bibha Choudhary Phone : 091 80 285289 00 extn. 105; Fax: 091 80 2852 8904

e-mail : vibha@ibab.ac.in

**Supplementary Materials**

**Supplementary Table 1. $IC_{50}$ values of various cancer cell lines upon treatment with SS28**. Values depicts the $IC_{50}$ value based on trypan blue assay at 48 h.

**Supplementary Figure 1. Evaluation of effect of SS28 on cell cycle progression. A.** A549 cells were treated with SS28 (5 µM) for different time points (6, 12, 18, 24 and 30 h) and cell cycle distribution was analyzed by flow cytometry after staining the cells with PI. The concentration selected was based on the $IC_{50}$ value. **B.** CEM cells were treated 2 µM of SS28 (6, 12, 18, 24 and 30 h) and cell cycle distribution was assessed by flow cytometry. In all panels DMSO treated cells served as vehicle control.

**Supplementary Figure 2. Evaluation of intracellular ROS production following treatment with SS28. A.** CEM cells treated with SS28 (5 µM) for different time points (5, 10, 15, 30, 60 min) and ROS generation was studied using flow cytometry analysis. $H_2O_2$ treated cells were used as positive control, while cells alone were used as negative control. DMSO treated cells were used as vehicle control. **B**. Histogram showing the ROS production is also presented.

| Cell lines | $IC_{50}$ (µM) |
|---|---|
| CEM | 2.6 |
| Reh | 7.9 |
| Molt4 | 5.1 |
| SUDHL8 | 2.7 |
| Nalm6 | 21 |
| A549 | 5.2 |
| 293T | 25.6 |
| HeLa | 32.4 |
| PBMC | 39.3 |
| MEF | 5.0 |

Supplementary Table 1

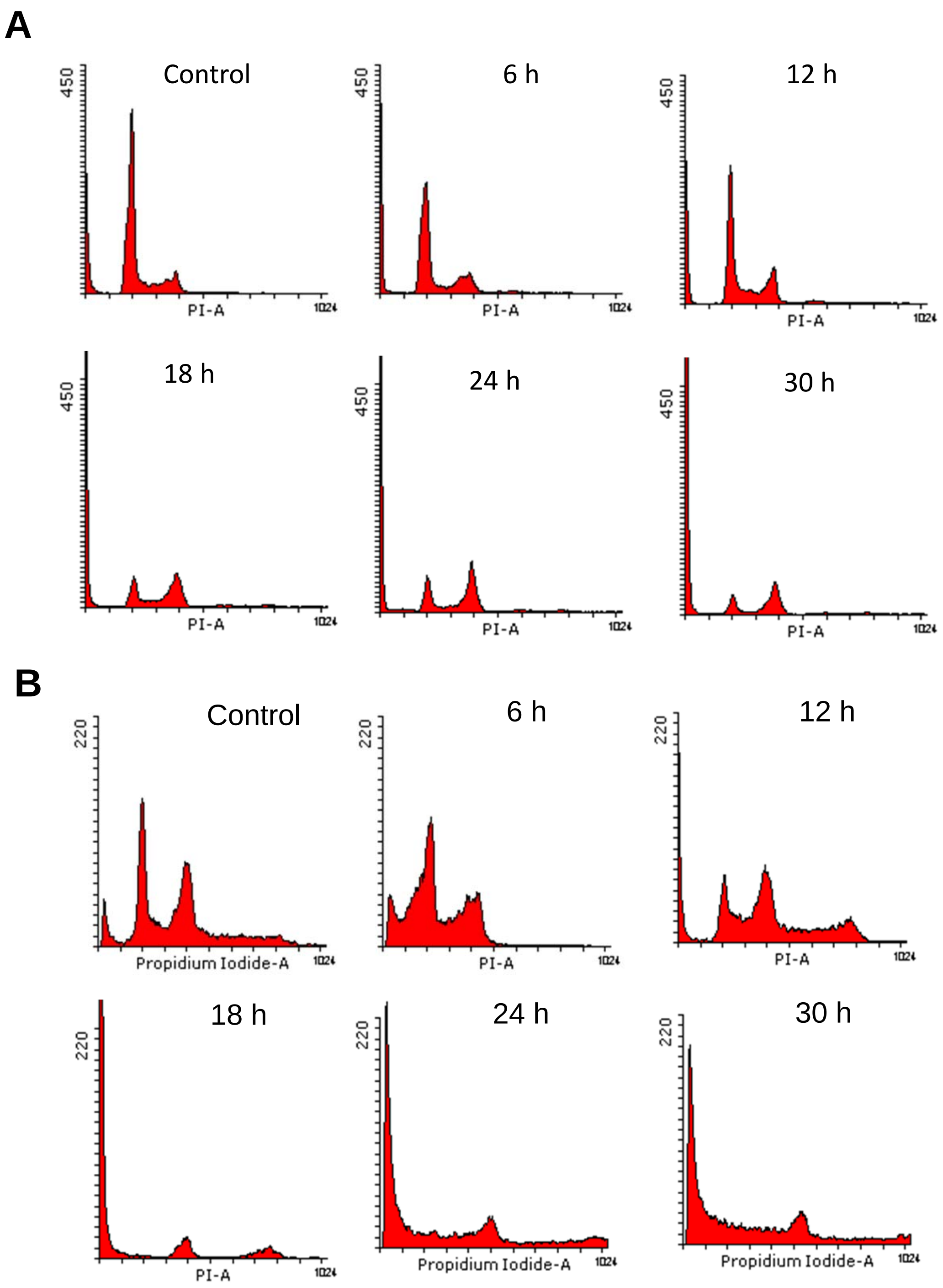


Supplementary Figure 1

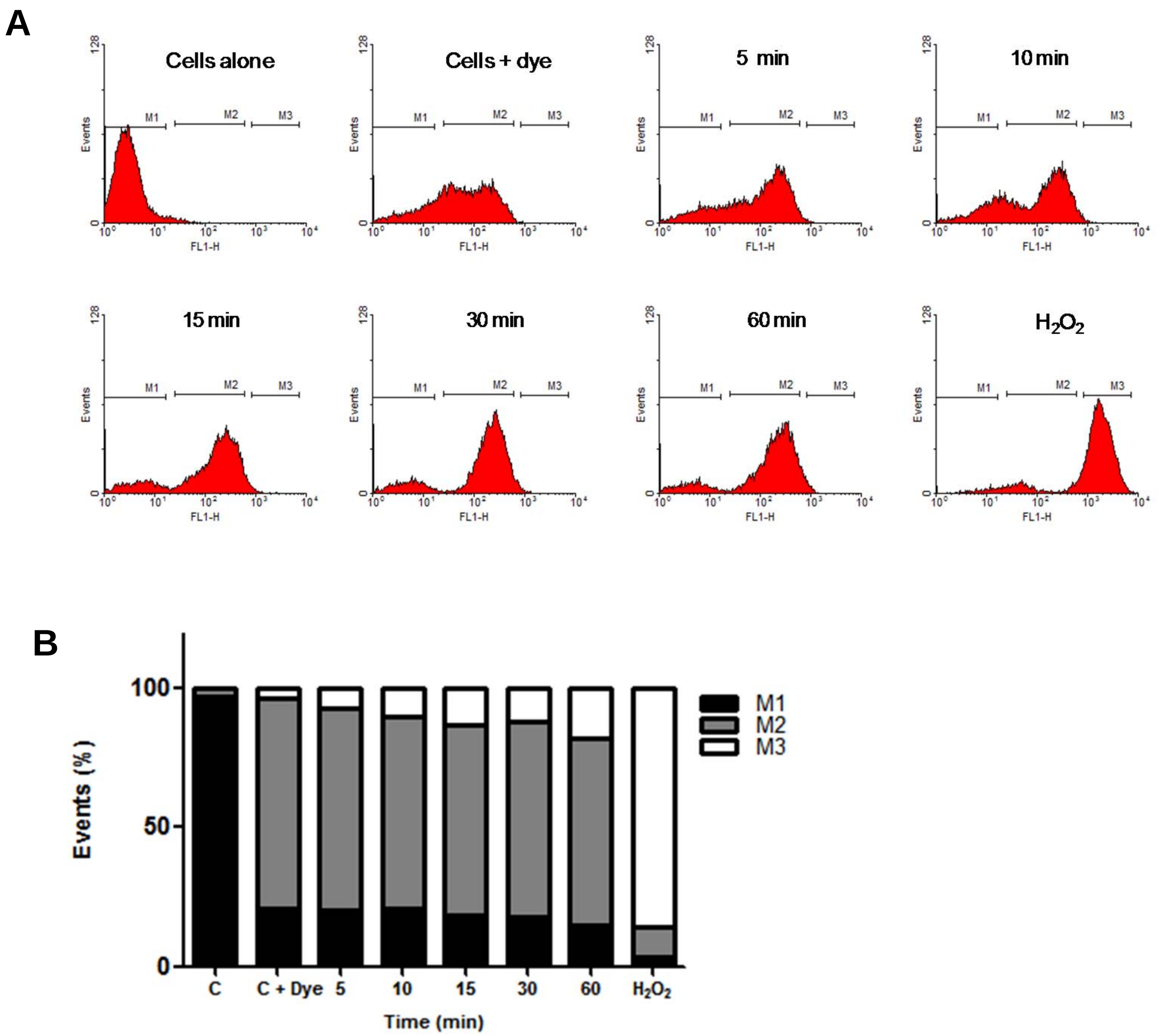


Supplementary Figure 2
